# Profound Asymptomatic Hyponatremia Associated With Anuric Acute Kidney Injury: Successful Controlled Correction Using Short Intermittent Hemodialysis

**DOI:** 10.7759/cureus.106293

**Published:** 2026-04-01

**Authors:** Ali El Khand

**Affiliations:** 1 Department of Nephrology, Faculty of Medicine and Pharmacy, Moulay Ismail University, Errachidia, MAR; 2 Department of Nephrology, Moulay Ali Cherif Regional Hospital Center, Errachidia, MAR

**Keywords:** acute renal failure, asymptomatic hyponatremia, hydro-electrolytic turbidity, intermittent hemodialysis, severe hyponatremia

## Abstract

Profound hyponatremia (<110 mmol/L) represents a critical electrolyte disturbance associated with significant neurological risk, particularly when rapid correction precipitates osmotic demyelination syndrome. Therapeutic management becomes especially complex when severe hyponatremia coexists with anuric acute kidney injury (AKI) requiring urgent renal replacement therapy, as intermittent hemodialysis may induce abrupt sodium shifts. We describe the case of a 66-year-old hypertensive woman receiving hydrochlorothiazide and perindopril who presented 10 days after cataract surgery with severe anuric AKI, profound asymptomatic hyponatremia (104 mmol/L), hyperkalemia (6.6 mmol/L), elevated creatinine (143 mg/L), and urea (2.6 g/L). Despite the severity of biochemical abnormalities, the patient remained conscious, hemodynamically stable, and neurologically intact, with normal thyroid and adrenal function. Given the urgency of treating hyperkalemia and uremia while avoiding overcorrection of sodium, a carefully individualized intermittent hemodialysis strategy was implemented using ultra-short initial sessions, controlled blood flow (220 mL/min), gradual dialysate sodium adjustment, and concomitant dextrose infusion. This approach enabled progressive correction of serum sodium to 126 mmol/L over four consecutive sessions without exceeding recommended safety thresholds and without neurological complications. This case highlights that meticulously tailored short-duration intermittent hemodialysis can achieve safe sodium correction while addressing life-threatening metabolic indications, suggesting a potential alternative when continuous renal replacement therapy is unavailable.

## Introduction

Hyponatremia is defined as a serum sodium concentration below 135 mmol/L and represents the most common electrolyte disorder encountered in clinical practice. Its clinical severity is not determined solely by the absolute sodium level but by the rapidity of onset and the presence of neurological symptoms. Indeed, a rapid decline in serum sodium may result in severe neurological manifestations even when levels are above 120 mmol/L [1-3].

Hyponatremia is classified as acute when it develops within less than 48 hours and chronic when it persists beyond 48 hours. This distinction is crucial, as acute hyponatremia carries a higher risk of cerebral edema due to the absence of adaptive mechanisms, whereas chronic hyponatremia is generally better tolerated.

In chronic hyponatremia, adaptive cerebral mechanisms lead to the loss of intracellular osmolytes, reducing brain swelling but increasing vulnerability to osmotic demyelination syndrome if correction occurs too rapidly [1-3]. The coexistence of severe hyponatremia and anuric acute kidney injury (AKI) presents a major therapeutic dilemma. Urgent renal replacement therapy may be required for hyperkalemia or uremia, yet intermittent hemodialysis can induce rapid increases in serum sodium concentration [4].

International guidelines recommend limiting sodium correction to 6-8 mmol/L per 24 hours or 4-6 mmol/L in high-risk patients [5-7]. Although continuous renal replacement therapy (CRRT) allows gradual correction, it is not always available. There are limited reports describing safe management strategies using short intermittent hemodialysis sessions in completely anuric patients.

We report a case of profound asymptomatic hyponatremia associated with anuric AKI successfully managed using a short intermittent hemodialysis strategy with careful sodium control.

## Case presentation

A 66-year-old woman with a history of hypertension treated with hydrochlorothiazide and perindopril, with no other known comorbidities, was admitted 10 days after cataract surgery for progressive deterioration of her general condition and anuria (<100 mL/24 hours). On admission, she was conscious, fully oriented, and neurologically intact with a Glasgow coma scale score of 15/15. Vital signs were stable, with a blood pressure of 135/80 mmHg, heart rate of 88 beats per minute, temperature of 36.8°C, and oxygen saturation of 98% on room air. There were no signs of confusion, seizures, focal neurological deficits, or respiratory distress. Careful clinical volume assessment revealed no peripheral edema, no ascites, no jugular venous distension, and no signs of dehydration. The patient was therefore considered clinically euvolemic at presentation.

Baseline renal function before admission was not available, and there was no documented history of chronic kidney disease. Initial laboratory investigations revealed profound hyponatremia with a serum sodium concentration of 104 mmol/L, severe hyperkalemia (6.6 mmol/L), markedly elevated creatinine (143 mg/L), and elevated urea (2.6 g/L), consistent with severe anuric AKI. Serum osmolality was calculated at 256 mOsm/kg, confirming hypotonic hyponatremia. Urine studies (urine sodium and urine osmolality) could not be performed due to complete anuria, as no urine sample could be obtained. Thyroid-stimulating hormone and morning cortisol levels were within normal ranges, excluding thyroid and adrenal insufficiency.

Preoperative serum sodium levels were not available, limiting the ability to determine whether hyponatremia was pre-existing. Detailed laboratory findings, including reference ranges, are presented in Table 1.

**Table 1:** Laboratory findings at admission TSH: Thyroid-stimulating hormone.

Parameters	Value	Reference Range
Serum sodium	104 mmol/L	135–145 mmol/L
Serum osmolality	256 mOsm/kg	275–295 mOsm/kg
Serum potassium	6.6 mmol/L	3.5–5.0 mmol/L
Serum creatinine	143 mg/L	6–12 mg/L
Urea	2.6 g/L	0.15–0.45 g/L
TSH	1.36 mUI/L	0.4–4.0 mIU/L
Cortisol at 8 am	412 nmol/L	140–690 nmol/L

The AKI was considered multifactorial. While chronic use of thiazide diuretics and angiotensin-converting enzyme inhibitors may have contributed, these alone do not fully explain the severity of the presentation. A more plausible mechanism is acute tubular necrosis precipitated by perioperative factors, including possible volume disturbances and hemodynamic changes, in the context of combined nephrotoxic medications.

Initial medical management of hyperkalemia, including cardiac membrane stabilization and intracellular potassium shifting strategies, was initiated. However, due to complete anuria, potassium elimination remained ineffective, and loop diuretics were not indicated.

Urgent intermittent hemodialysis was initiated due to severe hyperkalemia and uremic syndrome. Given the high risk of overly rapid sodium correction, a carefully controlled strategy was implemented using very short consecutive sessions with a low-flux membrane, controlled blood flow of 220 mL/min, gradual adjustment of dialysate sodium concentration, and concomitant dextrose infusion. Dialysis parameters and sodium evolution after each session are detailed in Table 2.

**Table 2:** Dialysis sessions and parameters

Session	Duration	Membrane	Dialysate Na (mmol/L)	Blood Flow	Infusion	Post-dialysis Na (mmol/L)
1st	40 min	Low-flux	130	220 mL/min	Dextrose solution	108
2nd	45 min	Low-flux	130	220 mL/min	Dextrose solution	113
3rd	1 h	Low-flux	133	220 mL/min	Dextrose solution	119
4th	2 h	Low-flux	135	220 mL/min	Dextrose solution	126

**Figure 1:**
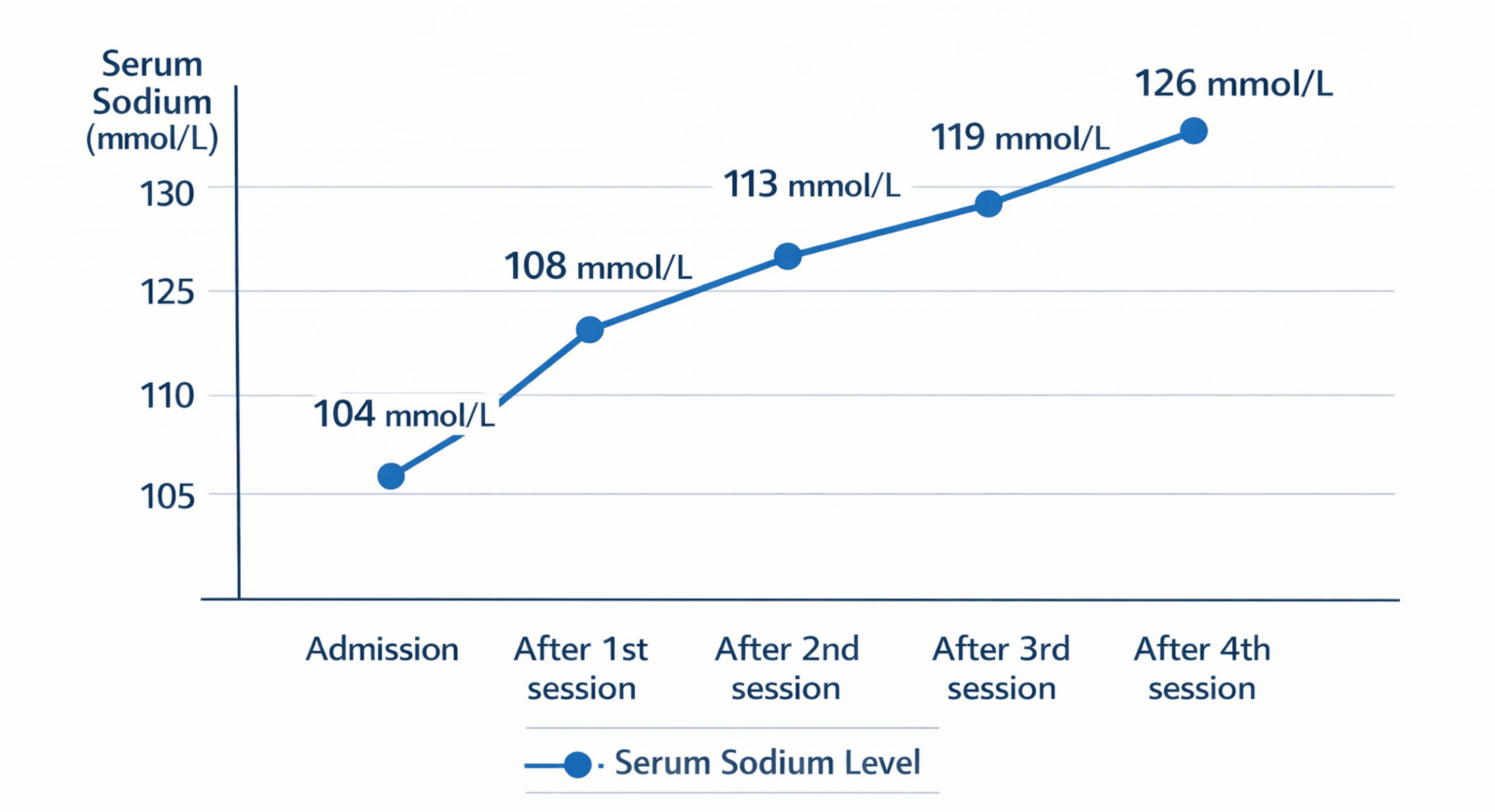
Evolution of serum sodium concentration over time during four consecutive intermittent hemodialysis sessions

As illustrated in Figure 1, serum sodium concentration increased progressively from 104 to 108 mmol/L after the first session, 113 mmol/L after the second, 119 mmol/L after the third, and 126 mmol/L after the fourth session. Daily correction remained within recommended safety limits, not exceeding 8 mmol/L per 24 hours. Close neurological monitoring was performed throughout hospitalization, and no neurological complications were observed.

A progressive improvement in potassium levels, urea concentration, and urine output was observed in parallel with dialysis sessions, supporting the effectiveness of the therapeutic strategy. At discharge, serum sodium reached 131 mmol/L, creatinine decreased to 20 mg/L, and urine output recovered to 1.5 liters per 24 hours, indicating progressive renal recovery.

## Discussion

Severe hyponatremia constitutes a critical metabolic emergency due to the potential for serious neurological complications, particularly when correction occurs too rapidly. In chronic hyponatremia, cerebral adaptation involves the gradual extrusion of intracellular osmolytes, which initially limits cerebral edema but renders the brain vulnerable to osmotic demyelination syndrome if serum sodium rises abruptly [1-4]. For this reason, current international guidelines recommend limiting sodium correction to 6-8 mmol/L within the first 24 hours and to 4-6 mmol/L in patients considered at high risk [5-7].

In the present case, multiple contributing factors were identified, including chronic thiazide therapy, postoperative stress likely associated with non-osmotic antidiuretic hormone secretion, and anuric AKI, preventing free water excretion [2,8]. The primary therapeutic challenge was the simultaneous need for urgent renal replacement therapy due to severe hyperkalemia and uremia in a context where intermittent hemodialysis carries an inherent risk of overly rapid sodium correction.

Our management strategy was based on very short initial intermittent hemodialysis sessions (40-45 minutes), controlled blood flow (220 mL/min), moderate and progressive adjustment of dialysate sodium concentration, and concomitant dextrose infusion. This individualized approach allowed gradual correction of serum sodium from 104 to 126 mmol/L over four consecutive sessions while remaining within recommended safety limits.

In this context, sodium correction was guided by recommended safety thresholds rather than predictive formulas. Given the absence of validated models for sodium kinetics during short intermittent hemodialysis in anuric patients, a pragmatic approach based on frequent biochemical monitoring and stepwise adjustment of dialysis parameters was adopted. Although formal sodium deficit calculations were not used to guide therapy, the observed sodium correction remained within expected safe limits based on estimated total body water. The use of relatively low dialysate sodium concentrations, combined with dextrose infusion, aimed to minimize the serum-to-dialysate sodium gradient and prevent rapid overcorrection.

Only limited data are available regarding the use of ultra-short consecutive intermittent hemodialysis sessions in completely anuric patients with profound hyponatremia. Although CRRT provides more precise control of sodium correction [9,10], it requires specialized equipment and intensive care resources that may not always be accessible. Our observation suggests that when continuous modalities are unavailable, carefully individualized intermittent hemodialysis can represent a safe, effective, and pragmatic alternative.

This case emphasizes the importance of individualized dialysis prescription, dynamic adjustment of dialysate sodium concentration, meticulous biochemical monitoring, and strict neurological surveillance when managing severe hyponatremia in patients requiring urgent dialysis.

## Conclusions

Profound hyponatremia associated with anuric AKI represents a complex therapeutic challenge, particularly when urgent dialysis is required. This case illustrates that progressive short-duration intermittent hemodialysis with individualized dialysate sodium adjustment can allow safe and controlled sodium correction while simultaneously addressing hyperkalemia and uremia. However, these findings should be interpreted with caution, given the single-case design. CRRT remains the preferred modality in this setting, and this approach may be considered a potential alternative in selected situations, particularly when CRRT is not available.
